# Rapid Intervention of Chlorpromazine and Promethazine for Hibernation-Like Effect in Stroke: Rationale, Design, and Protocol for a Prospective Randomized Controlled Trial

**DOI:** 10.3389/fneur.2021.621476

**Published:** 2021-03-17

**Authors:** Shuyu Lv, Wenbo Zhao, Gary B. Rajah, Chaitu Dandu, Lipeng Cai, Zhe Cheng, Honglian Duan, Qingqing Dai, Xiaokun Geng, Yuchuan Ding

**Affiliations:** ^1^Luhe Institute of Neuroscience, Capital Medical University, Beijing, China; ^2^Department of Neurology, Beijing Luhe Hospital, Capital Medical University, Beijing, China; ^3^China-America Institute of Neuroscience, Xuanwu Hospital, Capital Medical University, Beijing, China; ^4^Department of Neurosurgery, Wayne State University School of Medicine, Detroit, MI, United States; ^5^Department of Neurosurgery, Munson Medical Center, Traverse City, MI, United States; ^6^Department of Research and Development Center, John D. Dingell VA Medical Center, Detroit, MI, United States

**Keywords:** acute ischemic stroke (AIS), phenothiazine, neuroprotection, intravenous thrombolysis, rt-PA

## Abstract

**Background:** Following an acute ischemic stroke (AIS), rapidly initiated reperfusion therapies [i. e., intravenous thrombolysis (IVT) and endovascular treatment (EVT)] demonstrate robust clinical efficacy. However, only a subset of these patients can benefit from these therapies due to their short treatment windows and potential complications. In addition, many patients despite successful reperfusion still have unfavorable outcomes. Thus, neuroprotection strategies are urgently needed for AIS patients. Chlorpromazine and promethazine (C+P) have been employed in clinical practice for antipsychotic and sedative purposes. A clinical study has also shown a neuroprotective effect of C+P on patients with cerebral hemorrhage and subarachnoid hemorrhage. The safety, feasibility, and preliminary efficacy of intravenous administration of C+P in AIS patients within 24 h of onset will be elucidated.

**Methods:** A prospective randomized controlled trial is proposed with AIS patients. Participants will be randomly allocated to an intervention group and a control group with a 1:1 ratio (*n* = 30) and will be treated with standard therapies according to the current stroke guidelines. Participants allocated to the intervention group will receive intravenous administration of C+P (chlorpromazine 50 mg and promethazine 50 mg) within 24 h of symptom onset. The primary outcome is safety (mainly hypotension), while the secondary outcomes include changes in functional outcome and infarction volume.

**Discussions:** This study on Rapid Intervention of Chlorpromazine and Promethazine for Hibernation-like Effect in Stroke (RICHES) will be the first prospective randomized controlled trial to ascertain the safety, feasibility, and preliminary efficacy of intravenous C+P as a neuroprotection strategy in AIS patients. These results will provide parameters for future studies, provide insights into treatment effects, and neuroprotection with phenothiazine in AIS.

**Clinical Trial Registration:** www.chictr.org.cn, identifier: ChiCTR2000038727.

## Introduction

Due to its high rate of mortality and morbidity, ischemic stroke is a devastating public health concern which also results in a high socioeconomic burden (1, 2). Intravenous thrombolysis with recombinant tissue plasminogen activator (rt-PA) is an important method for the treatment of acute ischemic stroke. However, due to the narrow time window, disparate national health awareness, uneven distribution of medical resources, traffic congestion, and potential complications, only around 2.4% of patients receive this treatment currently in China (3). The mortality at 3–6 months after intravenous thrombolysis was not significantly reduced, and is as high as 17.9%, with 2/3 of the patients having varying degrees of disability (3–5). Therefore, attention must be given to both vascular recanalization and neuroprotection. Exploring fast and effective neuroprotection strategies to save time for recanalization strategies and improve prognosis will become a key strategy in the treatment of AIS. We believe chlorpromazine and promethazine (C+P) may benefit patients in the acute setting of AIS.

Hibernation is an altered physiological state during times of food shortage and is characterized by drastic decreases in both body temperature and metabolic rate. Animals in hibernation withstand the decline of cerebral blood flow without experiencing brain damage (6, 7). In acute ischemic stroke, the cascade reaction caused by ischemia leads to irreversible damage to the brain within minutes (8). If a hibernation-like state can be induced in AIS patients, it may reduce brain metabolism allowing the patient to reach a balance point between energy supply and demand. This may increase tolerance to ischemia and hypoxia. C+P are the two most widely used phenothiazine drugs to induce hibernation (9). C+P are both FDA-approved and routinely used in severe brain injury, brain edema, central high fever with restlessness, mental illness and delirium (9, 10). Existing data reveal that C+P is safe for most patients (11, 12). A previous clinical study found that a mixture of drugs, including chlorpromazine 50 mg + promethazine 50 mg + dolantin (pethidine) 100 mg, named as “lytic cocktail” was injected intravenously to produce sedation, analgesia, amnesia, hypotension, hypothermia, and blockade of the functions of the sympathetic and parasympathetic nervous systems during an event (9). The drugs reduced the mortality and neurological deficit of patients with acute cerebral hemorrhage and subarachnoid hemorrhage (13). Although a neuroprotective role was not investigated in acute ischemic stroke, the safety of an oral C+P regimen has been demonstrated by our group in stroke patients (14). In addition, our previous studies have demonstrated a neuroprotective effect of C+P in rat ischemic stroke models (15). The neuroprotective effect was due to the reduction in reactive oxygen species (ROS)-mediated oxidative injury (15), brain inflammation (16), apoptosis (17), and reduction in blood-brain barrier (BBB) dysfunction after ischemic stroke (18). Importantly, this neuroprotection was not completely dependent upon the reduction of body temperature. As a safe, effective, cost-efficient “old drug,” C+P may be repurposed as a promising new therapy to achieve neuroprotection in AIS patients. We thus designed this single-center, prospective randomized controlled study on Rapid Intervention of Chlorpromazine and Promethazine for Hibernation-like Effect in Stroke (RICHES) to verify the safety, feasibility, and preliminary efficacy of immediate intravenous administration of C+P at a dose as previously reported for AIS.

## Methods

### Study Design

This is a single-center, prospective randomized controlled trial. It will be performed in patients with acute ischemic stroke (AIS) onset within 24 h. Participants meeting inclusion criteria but not exclusion criteria will be randomly allocated to the C+P or the control group whether they received IVT or not. Participants allocated to the intervention group will receive intravenous administration of C+P within 24 h of symptom onset, within the first 60 min of administration. C+P will be administered after rt-PA if the patients received IVT. Seeing that early mechanical thrombectomy has an absolute difference in the rate of functional independence, as compared with alteplase alone (19, 20), this study will not include patients treated with EVT. *Via* the medical records, the demographic information, chronic medical history (diabetes, hypertension, hyperlipidemia, coronary heart disease), smoking history, alcohol drinking history, and NIHSS score at admission will be collected. Magnetic resonance imaging (MRI) will be performed at baseline and on days 3 and 90. NIHSS and mRS will be evaluated at baseline and immediately after administration, and on days 2, 3, 7, 14, and 90. All participants or proxies will be informed of potential risks and possible benefits and consented for this study. The consent will be provided to a legal representative if the patients do not have the capacity to consent. This study was approved by the ethics committee of Luhe Hospital, Capital Medical University, Beijing, China and has been registered at www.chictr.org.cn with ChiCTR2000038727. An independent physician will monitor the health and safety of the participants.

### Patient Population: Inclusion and Exclusion Criteria

Participants will be recruited from the stroke center (the Stroke Intervention and Translational Center, SITC) in Beijing Luhe Hospital. The inclusion criteria are (1) ≥18 and ≤80 years old; (2) clinical diagnosis of AIS; (3) NIHSS score ≥6 and ≤25; (4) patients with time from onset to treatment ≤24 hours that did not receive endovascular treatment (EVT); (5) pre-stroke mRS ≤2; (6) informed consent provided by participant or legally authorized representative.

Exclusion criteria are as follows: (1) rapid and spontaneous relief of symptoms: NIHSS score reduced to <6; (2) hemodynamic instability, including low systolic blood pressure (SBP) and SBP ≤100 mmHg despite standard treatment; (3) medical history of Parkinson's disease, Parkinson's syndrome, neuroleptic malignant syndrome, basal ganglia disease and epilepsy; (4) allergy to phenothiazines; (5) acute myocardial infarction, acute cardiac insufficiency and/or serious arrhythmia; (6) participant in another ongoing clinical trial; (7) life expectancy of fewer than 1 year due to comorbidities; (8) any condition which the investigator judges to increase the risk of injury to the patient.

### Randomization and Blindness

During the recruitment period, participants will be allocated 1:1 to two groups (*n* = 30) by computer-generated randomization procedures using opaque envelopes. A research assistant not involved in the study will prepare the envelopes before the study. After recording baselines measures, participants will be randomly allocated to either the intervention or the control group by the treating physicians who will open the sealed opaque envelopes. In order to minimize selection bias, patients and assessors involved in the trial will be masked to treatment allocation. All outcome measurements will be assessed by two observers blinded to the treatment plan, any disagreement will be resolved by reaching a consensus between the two. If no consensus can be reached, a third observer blinded to the treatment assignment and not involved in the clinical treatment plan will have a final decision. Finally, an independent investigator blinded to the treatment assignment will collect the data of outcomes and information of the group, and analyze them.

### Interventions

Participants in both groups will receive the standard management according to the guidelines for the treatment of ischemic stroke, including blood pressure control, treatment of hyperglycemia and hyperlipidemia, antiplatelet or anticoagulation, and neurotrophic treatment (21). The body temperature of both groups will be maintained at 36.0–37.3°C. The room temperature will be 20–25°C. In order to ensure the stability of administration speed and the stability of blood pressure, patients allocated to the C+P group will undergo intravenous infusion of chlorpromazine 50 mg and promethazine 50 mg at 5 mg/h. For the choice of drug dosage, we referred to the hibernation mixture (chlorpromazine 50 mg + promethazine 50 mg) (13). Based on clinical experiences, this dose is safe and effective. The same total dose of C+P (25 + 25 mg, twice a day) given to patients has been proven to be safe in our previous study. In addition, as compared to oral administration, the current protocol with continuous small dose injection at 5 mg/h *via* a micropump will maintain a stable blood concentration. The speed will be adjusted according to the patient's reaction. This administration will keep the patient in light sedation that can be awakened and allow for physical examination at any time. The control group will receive the same dosage of normal saline at 5 mg/h. During this period, vital signs (i.e., blood pressure, heart rate, body temperature, respiratory rate of the patients), changes in consciousness, and possible adverse drug reactions will be closely monitored. If the patients show a tendency to develop adverse reactions and complications, the trial shall be stopped immediately, and routine treatment shall be given.

### Outcomes

#### Primary Outcomes (Safety Assessment)

The main safety parameter is the incidence of hypotension requiring clinical intervention (≤90/60 mmHg). The secondary safety outcomes are the occurrence of adverse events including (1) neurologic deterioration defined as an increase of four or more points in the NIHSS within 7 days without clear explanation; (2) death during the study period regardless of cause; (3) body temperature down to below 36°C; (4) severe arrhythmias, such as sick sinus syndrome, rapid atrial fibrillation, supraventricular tachycardia, etc., which may cause palpitations, chest tightness, dizziness and other clinical manifestations; (5) liver damage: alanine aminotransferase (ALT) and aspartate aminotransferase (AST) concentrations were doubled; (6) seizures: epilepsy occurs within 2 weeks of stroke; (7) extrapyramidal responses (tremor, rigidity, salivation, and bradykinesia).

#### Secondary Outcomes (Efficacy Assessment)

The efficacy of the treatment is functional independence which will be assessed by the modified Rankin Scale (mRS, mRS scores of 0 to 2 indicating functional independence), and infarct volume as well as NIHSS scores. MRI will be performed at baseline and on days 3 and 90. We will measure the infarct volume on a Siemens Syngo with T1, T2, T2-flair, Diffusion-Weighted Imaging (DWI), Arterial Spin Labeling (ASL) and Intra-Voxel Incoherent Motion (IVIM). We will calculate the infarct volume by using the image tool (ROI) on the workstation to outline the lesion on each slice to compute the area, and multiply the area of each layer by the thickness of each layer.

### Estimation of Sample Size

There is no data available for reference because no completed clinical study of C+P in AIS patients without EVT currently exists. However, Hertzog has suggested that 10 to 20 patients in each group are sufficient to assess the feasibility of a pilot study (22) while Dobkin has shown that 15 patients in each group is usually enough to decide whether a larger multicenter trial should be conducted (23). Therefore, our goal is to recruit 15 patients in each group, a similar number of samples was selected for another protocol (24). The results of this study should be able to determine the initial safety and feasibility of intravenous infusion of C+P in AIS patients. The data will be used to estimate sample size and conduct a power calculation to plan a phase-2 trial for efficacy.

### Statistical Analyses

The endpoint event analysis is based on the intention-to-treat (ITT) principle, including all randomly enrolled subjects. If compared to the control group, the treatment group did not have an increase in the incidence of adverse reactions and there was no difference in 90 days prognosis between the two groups, we would move forward with a phase-2 trial.

Categorical variables including the proportion of good functional outcomes and the frequency of adverse events will be presented as counts and percentages. χ^2^-test, Fisher exact-test, or continuity correction will be used where appropriate for comparison between the two groups. If the continuous variables including NIHSS score and infarct volume conform to the normal distribution, it shall be indicated by mean ± standard, and tested by *t*-test, and if it does not conform to the normal distribution, it shall be indicated by median and interquartile range and tested by Mann-Whitney *U*-test. *p* < 0.05 will be considered statistically significant. SPSS 22.0 software will be used for statistical analysis.

## Discussion

A previous clinical study found that “lytic cocktail” [chlorpromazine 50 mg + promethazine 50 mg + dolantin (pethidine) 100 mg] reduced the mortality and neurological deficit of patients with acute cerebral hemorrhage and subarachnoid hemorrhage (13). Pethidine is mainly used for traumatic pain with acute cerebral hemorrhage and subarachnoid hemorrhage. Patients with acute ischemic stroke usually do not need analgesia, so in this study, we focused on the neuroprotective effect of C+P, without pethidine. Although a neuroprotective role was not investigated in acute ischemic stroke, our clinical study has demonstrated that the orally taken C+P at a dose of 12.5 + 12.5 mg or 25 + 25 mg, twice a day after AIS was safe by determining no serious adverse effects such as extrapyramidal symptoms, hypotension, seizures, abnormal liver function, ECG abnormality, and allergic reaction as well as NIHSS score and mRS in the treatment group compared with control group (14). Considering the low bioavailability of oral drugs, unstable blood drug concentration and other factors, the purpose of the present study will be to determine the safety, feasibility, and preliminary efficacy of the RICHES protocol. C+P will be intravenously administered, in order to establish a novel effective neuroprotective strategy. Our studies in rat ischemic stroke models demonstrated a neuroprotective effect of C+P. This protection was due to the reduction in the reactive oxygen species (ROS)-mediated oxidative injury (15), reduction in brain inflammation determined by the expression of TNF-α, IL-1 β, ICAM-1, VCAM-1, NF-κB (16), and reduction in apoptotic signal cascade (17), as well as improved integrity of blood-brain barrier (18). Importantly, the neuroprotection was induced in both hypothermic or normothermic conditions after ischemic stroke. In order to develop a translational strategy of neuroprotection, we will perform this study utilizing an intravenous infusion of C+P as a possible stable and effective therapy for AIS patients.

With an aging population, the incidence of stroke will increase significantly (1, 25, 26). Good prognosis of AIS patients depends on timely revascularization and effective neuroprotective agents. At present, the effective methods of revascularization are thrombolysis and thrombectomy. However, the clinical efficacy of these therapies is limited by the narrow time window, potential complications and reperfusion injuries such as hemorrhagic transformation and brain edema (3, 27–29). Exploring fast and effective neuroprotective strategies to earn time for vascular recanalization and reduce reperfusion injuries has become an essential desire in AIS treatment.

Over the past few decades, there have been over 1,000 neuroprotective methods that have been examined for potential neuroprotective properties, mild hypothermia is the only one proven effective in randomized controlled trials. Although not included in the guidelines, the National Institutes of Health (NIH) points out that mild hypothermia is the most promising neuroprotective method for ischemic stroke. However, hypothermia is difficult to establish, slow in cooling and can easily lead to serious complications such as arrhythmia and pulmonary infection. This makes it difficult to achieve the clinical translation of benefits (21, 30, 31). During hibernation, the cerebral blood flow is decreased by <10%, but the utilization of glucose in the brain decreases by 98%, uncoupling metabolic demands (32). Therefore, an induced hibernation-like state might be a potential therapy for ischemic stroke by maintaining the body's low metabolism and low oxygen consumption even without low body temperature. This state may help the body survive prolonged hypoxia during the “difficult period of AIS.”

C+P, as the main component of the classic prescription “lytic cocktail,” are the two most studied phenothiazine drugs (9). Based on their sedative and antipsychotic effects, they are widely used in severe brain injury, brain edema and schizophrenia since the 1950s (10). Existing data have demonstrated their clinical safety (11, 12). Chlorpromazine has been shown to protect against ischemic injury in liver, kidney, spinal cord and brain (33–39), promethazine can reduce the oxygen consumption and basic metabolic rate of the brain and whole body, as well as improve the tolerance of a tissue to hypoxia (40). In our animal studies, C+P has been found to reduce infarct volumes (from 50.3 to 35.7%, *p* < 0.05) and neurological deficits (from score 3.5 to 2.8, *p* < 0.05) in 2 h middle cerebral artery occlusion models. This appears most likely independent of drug-induced systemic hypothermia (15). In clinical practice, as mentioned above, oral administration of C+P after AIS was safe and there were no adverse reactions (14). Therefore, C+P application in stroke seems to have several advantages, such as simple implementation, rapid onset, cost-efficient, minimal known serious side effects in a small study. In addition, it could ultimately be administered in the field prior to reperfusion for neuroprotection and time window extension.

There are limitations to this study. First, this is a single-center, small sample experiment, which may affect the generalizability of the interventions and opens the study up to spurious results. Second, the target dosage has been shown to be safe and reliable in other small cohort experiments, but the dose used in the present study may still need optimization. In addition, there is a concern that an intravenous administration of an equal dosage might have more interactions given better bioavailability. The current study protocol will not include AIS patients who receive EVT to avoid interactions between C+P and moderate sedation or general anesthesia.

The RICHES study is designed to identify the safety, feasibility, and secondarily any possible efficacy of intravenous administration of C+P in AIS patients. The preliminary results will provide clues for the design of future clinical trials. If there is no significant difference in the incidence of drug-related adverse events between the experimental group and the control group, further clinical trials will be indicated. Based on past basic research and previous clinical studies, we predict that chlorpromazine and promethazine is safe for patients with acute ischemic stroke. The current study may suggest a neuroprotective role in AIS, and warrant a randomized controlled trial (RCT).

## Ethics Statement

The studies involving human participants were reviewed and approved by the Ethics Committee of Luhe Hospital, Capital Medical University, Beijing, China. The patients/participants provided their written informed consent to participate in this study.

## Author Contributions

SL, WZ, LC, ZC, HD, and QD prepared the manuscript. YD and XG designed the study. YD, GR, WZ, and CD revised the manuscript. All authors reviewed the manuscript and provided the final approval for the manuscript.

## Conflict of Interest

The authors declare that the research was conducted in the absence of any commercial or financial relationships that could be construed as a potential conflict of interest.
